# Bioreactors for high cell density and continuous multi-stage cultivations: options for process intensification in cell culture-based viral vaccine production

**DOI:** 10.1007/s00253-015-7267-9

**Published:** 2016-01-13

**Authors:** Felipe Tapia, Daniel Vázquez-Ramírez, Yvonne Genzel, Udo Reichl

**Affiliations:** International Max Planck Research School for Advanced Methods in Process and Systems Engineering, Sandtorstr. 1, 39106 Magdeburg, Germany; Max Planck Institute for Dynamics of Complex Technical Systems, Sandtorstr. 1, 39106 Magdeburg, Germany; Chair for Bioprocess Engineering, Otto-von-Guericke-University Magdeburg, Universitätsplatz 2, 39106 Magdeburg, Germany

**Keywords:** Viral vaccine production, Process intensification, Perfusion, Fed-batch, Feeding strategy, Two-stage bioreactor, Continuous cultivation, Passage effect

## Abstract

With an increasing demand for efficacious, safe, and affordable vaccines for human and animal use, process intensification in cell culture-based viral vaccine production demands advanced process strategies to overcome the limitations of conventional batch cultivations. However, the use of fed-batch, perfusion, or continuous modes to drive processes at high cell density (HCD) and overextended operating times has so far been little explored in large-scale viral vaccine manufacturing. Also, possible reductions in cell-specific virus yields for HCD cultivations have been reported frequently. Taking into account that vaccine production is one of the most heavily regulated industries in the pharmaceutical sector with tough margins to meet, it is understandable that process intensification is being considered by both academia and industry as a next step toward more efficient viral vaccine production processes only recently. Compared to conventional batch processes, fed-batch and perfusion strategies could result in ten to a hundred times higher product yields. Both cultivation strategies can be implemented to achieve cell concentrations exceeding 10^7^ cells/mL or even 10^8^ cells/mL, while keeping low levels of metabolites that potentially inhibit cell growth and virus replication. The trend towards HCD processes is supported by development of GMP-compliant cultivation platforms, i.e., acoustic settlers, hollow fiber bioreactors, and hollow fiber-based perfusion systems including tangential flow filtration (TFF) or alternating tangential flow (ATF) technologies. In this review, these process modes are discussed in detail and compared with conventional batch processes based on productivity indicators such as space-time yield, cell concentration, and product titers. In addition, options for the production of viral vaccines in continuous multi-stage bioreactors such as two- and three-stage systems are addressed. While such systems have shown similar virus titers compared to batch cultivations, keeping high yields for extended production times is still a challenge. Overall, we demonstrate that process intensification of cell culture-based viral vaccine production can be realized by the consequent application of fed-batch, perfusion, and continuous systems with a significant increase in productivity. The potential for even further improvements is high, considering recent developments in establishment of new (designer) cell lines, better characterization of host cell metabolism, advances in media design, and the use of mathematical models as a tool for process optimization and control.

## Introduction

Most biologicals produced in animal cell culture are continuously synthesized during the cell proliferation phase. Recombinant proteins, for example, are typically produced in batch or fed-batch mode, where the product is accumulated in the culture broth and harvested once peak concentrations are reached (Castilho et al. 2008). In contrast, the production of viral vaccines typically requires a cell growth phase followed by a virus replication phase (both typically operated in batch mode) as most viruses propagate in a complex process that requires the internalization of their genetic material into the host cell, the synthesis of viral RNA/DNA and viral proteins as well as the release of progeny virus particles (Aunins 2003). Furthermore, it has to be taken into account that the replication process of lytic viruses results in cell death due to apoptosis followed by cell degradation and release of contaminants such as cellular DNA and host cell proteins.

To increase virus production yields through process optimization, three key factors need to be considered:

- Cell concentration and metabolic/physiological status of the cells at time of infection (toi): As a general rule, cell concentration defines final virus titers. However, it is essential to perform infections on healthy cells, with no limitation of key nutrients, not inhibited by accumulated by-products such as lactate and ammonia, and in an appropriate growth status (i.e., dividing/non-dividing, cell cycle phase) (Demarchi and Kaplan 1977).
- Ratio of infectious particles to viable cells, namely multiplicity of infection (moi) at the time of infection (toi): Since virus transport to the target cell in the culture medium is governed by diffusion, an optimal amount of virus particles per cell should be inoculated to counteract degradation/inactivation of infectious virions before they reach their host cell (Aunins 2003). In addition, for most viruses, a too high number of virus particles per cell at toi can promote replication of so-called defective interfering particles (DIPs), which decreases maximum virus yields. This is of particular importance in continuous cultivations using cascades of stirred tank bioreactors (STR), where decreases in virus titers for long cultivation times have been observed as a consequence of DIP accumulation (Frensing 2015; van Lier et al. 1990).
- Residence time (RT) of virus particles within the bioreactor and time point of harvest (toh): The RT can be defined as the time that a cell or a virus particle remains inside the bioreactor and is characteristic for the cultivation mode. In closed systems operated in batch cultivation mode, the RT is identical for all particles and equivalent to the harvest time. After having achieved maximum titers, virus infectivity as well as the total number of virus particles can decrease again (Aunins 2003), while the extracellular DNA and protein contamination level can increase significantly due to cell lysis. Accordingly, time of harvest has to be determined carefully, taking into account vaccine type (live attenuated, inactivated) and downstream processing requirements. In particular, for viral vaccines, where potency depends totally or partially on infectivity (e.g., live attenuated vaccines, viral vectors), a short RT/early toh is beneficial. When batch knowledge is transferred to continuous systems, the picture is more complex as not all particles spend the same time inside the continuously operated bioreactor. In continuous bioreactors, an important concept is the RT distribution, which is essentially a statistical approach to describe the probability of particles to leave the bioreactor (Levenspiel 1972; Sarkar et al. 2015). Nevertheless, a good approximation is given by the average RT that, in continuous STRs, equals the inverse of the dilution rate (RT = 1/D).

**Fig. 1:**
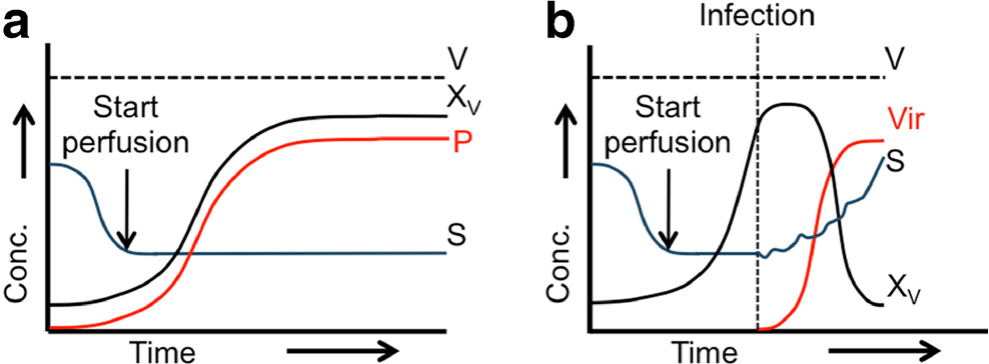
Schematic representation of the perfusion-based high cell density (HCD) production of recombinant proteins and viruses. **a** Concentration profiles of different performance parameters in a perfused bioreactor for the production of recombinant proteins. **b** Concentration profiles of different performance parameters in a perfused bioreactor for the production of viruses. *V* volume, *X*_*V*_ cell concentration, *P* recombinant protein concentration, *Vir* virus particle concentration, *S* substrate (glucose) concentration

In order to address the impact of the first key factor (cell concentration, physiological status) on process intensification, approaches towards optimization of upstream processes for manufacturing of other biologicals, i.e., CHO cell-derived recombinant proteins, can serve as a general guideline. Here, high cell density (HCD) processes have been developed for the production of biopharmaceuticals over more than 20 years (Kompala and Ozturk 2006; Ozturk 1994, 1996), and a high number of products have been introduced into the market (Kompala and Ozturk 2006; Pollock et al. 2013). Typically, HCD processes rely on fed-batch or perfusion strategies and the use of one or the other depends on specific requirements of the product and technical aspects. However, perfusion bioreactors have the potential to achieve higher cell concentrations since they offer a constant nutrient-enriched environment avoiding the accumulation of unwanted by-products (Fig. 1a) (Castilho et al. 2008). In addition, these cultivation conditions allow the use of cultivation systems with a low footprint and with high volumetric production rates (Ozturk 1994, 1996). For process intensification in viral vaccine production, however, significant differences compared to the classical production process of recombinant proteins exist. Due to the separation of most of the virus production processes in a cell growth phase and a virus replication phase, different production profiles and kinetics are to be expected (Fig. 1b). In particular, the use of specific process strategies during virus propagation needs to be considered.

Regarding the cell retention in perfusion systems, a large variety of examples can be found for the production of recombinant proteins (Pollock et al. 2013). In general, filtration-based systems (i.e., internal and external spin filter, ATF and TFF), gravity settlers, and acoustic filters have been extensively used in the industry as well as in academia (Clincke et al. 2013; Kompala and Ozturk 2006; Pollock et al. 2013). Some of these systems have potential drawbacks such as filter clogging (membrane-based systems) and limited scalability (gravity settlers and acoustic filters). Accordingly, alternating tangential flow (ATF) and tangential flow filtration (TFF) systems have attracted considerable attention since they have a reduced risk of filter clogging (due to the cross-flow filtration) and can be easily scaled up based on the surface area of the hollow fiber cartridge (Kompala and Ozturk 2006). All this allows cultivations to very high cell concentrations in the order of 10^8^ cells/mL (Clincke et al. 2013). Other options for HCD cultivation of animal cells are fixed-bed reactors and entrapping retention systems. These systems, however, are known for heterogeneities regarding the distribution of medium components and gases (de la Broise et al. 1992) as well as for their operational complexity (Kompala and Ozturk 2006). Nevertheless, a recently developed fixed-bed bioreactor (CellTank®, PerfuseCell) has shown homogeneous concentration of metabolites allowing cultivation of CHO K1 cells at concentrations up to 2 × 10^8^ cells/mL (Zhang et al. 2015).

Process yields of viral vaccine manufacturing can also be improved using true continuous systems, i.e., chemostats (Kilburn and van Wezel 1970) or multi-stage systems such as the two-stage bioreactor (Frensing et al. 2013). Continuous bioreactors can operate at steady-state conditions (constant cell and metabolite concentration, pH value, and osmolality) avoiding shutdown times for cleaning and sterilization cycles typical for batch operation (Hoskisson and Hobbs 2005; Konstantinov and Cooney 2015). It was estimated that continuous cultivations can reduce operational costs by up to 55 % compared to batch processes (Walther et al. 2015). Manufacturing of biologicals at steady-state conditions is also assumed to positively influence the quality of the final product as, e.g., more consistent glycoform profiles and reduced protein deamidation can be obtained (Konstantinov and Cooney 2015). If viral vaccines are produced, steady-state operation at optimum virus RT could help to prevent fast degradation of infectious virus particles compared to batch cultivations, being beneficial for production of live-attenuated vaccines or viral vectors such as MVA virus (Jordan et al. 2013). The main problem with the use of true continuous cultivation strategies in viral vaccine production is, however, that it is not clear whether vaccines produced would be acceptable for regulatory agencies such as FDA and EMA as several open questions remain to be answered regarding the long-term genetic stability of cell substrates and virus strains (Gallo-Ramirez et al. 2015). In particular, it has to be investigated in detail, whether undesired viral mutations can accumulate overproduction time that can negatively influence potency and safety of vaccines. Similarly, in continuous systems, moi can increase during cultivation time as a consequence of virus replication and therefore promote excessive production of DIPs as addressed above (Frensing 2015). Finally, there is still little experience regarding more complex aspects of the viral life cycle (i.e., virus latency, lytic stages) on virus yields and on vaccine quality (Kilburn and van Wezel 1970; Roumillat et al. 1980).

In the following, we present a comprehensive overview of options for process intensification in cell culture-based viral vaccine production. In particular, we consider the establishment of HCD cultivations and the use of continuous multi-stage bioreactors. The focus of HCD will be on fed-batch strategies and operation in perfusion mode using ATF systems, hollow fiber bioreactors and acoustic filters as these systems have the highest potential for production of viral vaccines. In addition, process options regarding the use of two- and three-stage continuous bioreactors for virus production are addressed. Literature regarding baculovirus-insect cell expression systems is highlighted here due to its significant contribution to the understanding of virus dynamics in continuous cultures. As very few studies deal with HCD cultivations or continuous production systems in large-scale virus vaccine manufacturing, mainly, results obtained at laboratory scale will be presented for illustration of process options.

## Virus production at high cell densities

The term “high cell density” was previously defined as any cell concentration in the order of 10^7^ cells/mL (Griffiths et al. 1992). However, authors have often taken as a reference the typical cell concentrations achieved so far for a reference production process (typically batch) for that specific cell line. For many of the conventional cell lines used in vaccine production, this was in the range of 2 × 10^6^ cells/mL to 4 × 10^6^ cells/mL. Based on that, cell concentrations one order of magnitude higher than those obtained by established cultivation processes have been considered as high cell densities.

Given the nature of most virus propagation processes, where cells are infected at the late exponential growth phase, the cell concentrations need first to be increased to concentrations that cannot be achieved in batch mode for a HCD process. In addition, virus propagation at such high cell concentrations must be performed at the optimal conditions to avoid the so-called cell density effect, which is a reduction in the cell-specific virus yield (Lindsay and Betenbaugh 1992; Maranga et al. 2003). A summary of HCD virus production processes and their main characteristics is given in Table 1.

**Table 1:** Overview on viruses produced in high cell density cultures reported in literature

Virus	Cell line	Type	Maximum cell concentrations (×10^6^ cell/mL)	Bioreactor type	Cell proliferation	Virus infection/propagation	Virus harvest during propagation	Highest yields	Comments	Reference
Adenovirus	HEK293	Suspension	8	STR/TFF	Perfusion	Halting/perfusion	No	7.8 × 10^9^ IVP/mL at 35 °C	5.5 times higher than the batch control in spinner flask	Cortin et al. 2004
Adenovirus ONYX-411 (recombinant oncolytic vector)	HeLaS3 human tumor cell	Suspension	14.8	STR/TFF	Perfusion	Halting/perfusion	No (intracellular viral vectors)	6 × 10^11^ VP/mL	Titer sevenfolds higher than those achieved in fed-batch	Yuk et al. 2004
Adenovirus type 5	HEK293	Suspension	3	STR/acoustic settler	Perfusion	Perfusion	No (intracellular viral vectors, high MOI = 20 at infection)	16,200 VP/cell 6.0 × 10^9^ VP/mL	Cell-specific and volumetric yields comparable to batch. However, infection at a cell density of 6 × 10^6^ cells/mL led to a fivefold reduction in specific productivity	Henry et al. 2004
Adenovirus type 5	HEK293	Suspension	7.8	STR/TFF	Perfusion	Halting/perfusion	No	4.4 × 10^10^ IVP/mL 5600 IVP/cell	Cell-specific yield 4.3 times higher than batch	Gálvez et al. 2012
Adenovirus type 5, 26, 35	PER.C6	Suspension	16	STR/TFF	Perfusion	Perfusion	No (intracellular viral vectors)	50,000–150,000 VP/cell 1.0 × 10^12^ rAd26 virus particles VP/mL	Ratio VP/IVP of 20:1	Van and Luitjens 2011
A/PR/8/34 influenza (H1N1) virus	MDCK	Adherent	6.2	STR	Repeated fed-batch	Repeated fed-batch	Discontinuous	13,630 VP/cell 5248 HA/100 μL	Cell-specific and volumetric yields higher than batch	Bock et al. 2011
A/PR/8/34 influenza virus	HEK293	Suspension	6	STR/acoustic settler	Perfusion	Perfusion	Continuous	3960 VP/cell	Cell-specific yield increased fourfolds	Petiot et al. 2011
A/New Caledonia/20/99 influenza (H1N1) virus	MDCK	Adherent	8	Fixed bed	Recirculation	Recirculation	No, washout with PBS and medium exchange before infection	2.89 log_10_ (HA units/100 μL) and 7.8 × 10^7^ TCID50/mL	Novel disposable pack-bed bioreactor	Sun et al. 2013
A/PR/8/34 influenza (H1N1) virus	CAP	Suspension	26.9	STR/ATF	Perfusion	Perfusion	No	4086 VP/cell 1.18 × 10^12^ VP/d L	Cell-specific and volumetric productivity comparable to batch	Genzel et al. 2014
A/PR/8/34 influenza (H1N1) virus	AGE1.CR	Suspension	28.1	STR/ATF	Perfusion	Perfusion	No	1708 VP/cell 7.0 × 10^11^ VP/d L	Cell-specific and volumetric productivity comparable to batch	Genzel et al. 2014
A/PR/8/34 & A/Mexico/4108/2009 influenza (H1N1) virus	MDCK	Adherent/Suspension	40/28	Hollow fiber	Recirculation	Periodic harvest	Discontinuous	A/PR/8/34 (in suspension MDCK cells): 19,138 VP/cell 2.64 × 10^11^ VP/d A/Mexico/4108/2009 (in suspension MDCK cells): 3219 VP/cell	Cell-specific yields comparable to previous reports in batch	Tapia et al. 2014
Moloney murine leukemia virus (MoMLV) retrovirus vector	293GPG	Suspension	11	STR/Acoustic settler	Perfusion	Perfusion	Continuous	3–4 107 IVP/mL	Twentyfold increase in specific productivity compared to adherent cells. Cell line produces infective virus constitutively, no need of infection	Ghani et al. 2006
*Parapoxvirus ovis*	BK	Adherent	7	STR	Periodic medium exchange	Volume expanded fed-batch	No	1.06 × 10^8^ VP/Ld	Twentyfold increase in volumetric productivity, compared to batch	Pohlscheidt et al. 2008
Poliovirus (PV) type 1, 2 & 4	Vero	Adherent	2	STR	Semi-batch	Fed-Batch (Glucose/Gln)	No	356 DU/mL (PV1)	1.5- to 2-fold increase in cell-specific and volumetric yield compared to batch	Thomassen et al. 2014
Rabies	Vero	Adherent	5	STR/spin filter	Recirculation	Perfusion	Continuous	1.38 × 10^8^ FFU/mL	2.6-fold higher specific productivity than batch	Rourou et al. 2007

*VP* total viral particles, *IVP* infective viral particles, *FFU* fluorescent focus units, *DU* D-antigen units, *HA* hemagglutinin

### Process options to achieve high densities for adherent and suspension cells

It is important to note that maximum cell concentrations that are typically achieved differ between adherent and suspension cells. Provided similar aeration conditions, the maximum number of adherent cells that can be obtained in bioreactors depends on the available growth surface, while the maximal growth of suspension cells is mainly limited by the total amount of nutrients in the growth medium and the accumulation of growth-inhibiting compounds. In addition, further limitations for HCD processes using suspension cells such as space, reactor design, and operation as well as aeration have to be considered as discussed previously (Ozturk 1996). In particular, the addition of medium (fed-batch) or the exchange of medium (perfusion) is usually not sufficient for obtaining HCD with adherent cells, and an increase in growth surface, for example, by the addition of microcarriers in STRs, is required. The use of microcarriers offers the additional advantage that it allows an easy exchange of medium by sedimentation of carriers after switching off the stirrer of cultivation vessels or stopping the rocking unit of wave systems. For instance, an increase from 1.8 × 10^6^ cells/mL to 1.1 × 10^7^ cells/mL could be achieved for adherent Madin-Darby canine kidney (MDCK) cells when increasing the concentration of the microcarrier Cytodex 1 from 2.0 to 12.5 g/L using a repeated fed-batch process (Bock et al. 2011). Medium exchange was performed based on an estimated cell-specific feeding rate (Dowd et al. 2003). In another example, the proliferation of bovine kidney (BK) cells on Cytodex 3 for the propagation of *Parapoxvirus ovis* up to 7.0 × 10^6^ cells/mL was carried out using a periodic medium exchange (Pohlscheidt et al. 2008) based on the minimum glucose concentration measured. Finally, a recirculation-based feeding mode was applied for the propagation of Vero cells grown on microcarriers at about 6.0 × 10^6^ cells/mL for subsequent infection with various poliovirus serotypes (Thomassen et al. 2014). In this process, a fresh medium of an STR was circulated through the cultivation bioreactor at increasing rates depending on cultivation time.

Although similar to the recirculation strategy followed by Thomassen et al. (2014), a special case has been reported for the proliferation of both adherent and suspension MDCK cells in a single-used hollow fiber bioreactor for propagation of pandemic influenza virus (Tapia et al. 2014). Here, cell concentrations of about 3.0 × 10^7^ cells/mL were obtained by recirculation of fresh medium through the hollow fibers providing nutrients to the cells and diluting accumulated toxic compounds.

Besides adherent cell lines, suspension cells are routinely grown to HCD using fed-batch (Xie and Wang 1994) and perfusion-based feeding strategies (Kompala and Ozturk 2006). For example, the use of perfusion systems in the production of adenoviral vectors has been described by Nadeau and Kamen (2003). However, cell concentrations did not exceed 6.0 × 10^6^ cells/mL and neither a significant change in volumetric yield nor in cell-specific yields was observed compared to batch cultivations. Since this application has been comprehensively described by Nadeau and Kamen (2003), it will not be addressed further in this review. More recently, external cell retention systems such as acoustic filters (Petiot et al. 2011) or the ATF system (Genzel et al. 2014) have been used in various vaccine production processes established in research laboratories. Using an acoustic filter, suspension HEK293 cells have been grown to concentrations approaching 6.0 × 10^6^ cells/mL before infection with a recombinant adenovirus type 5 (Henry et al. 2005) and type A influenza virus (Petiot et al. 2011), respectively. In this case, cell growth continued even after infection reaching 11 (Henry et al. 2005) and 14 × 10^6^ cells/mL (Petiot et al. 2011). In another study, the designer cell lines AGE1.CR and CAP have been cultivated to 4.8 and 3.3 × 10^7^ cells/mL, respectively, for the propagation of type A influenza virus (Genzel et al. 2014; Villiger-Oberbek et al. 2015) using an ATF system. Significant efforts have also been reported regarding options to intensify vaccine production processes using PER.C6 cells. Although this cell line can be cultivated up to 1.0 × 10^7^ cells/mL in batch (Sanders et al. 2013) and above 1.0 × 10^8^ cells/mL in perfusion mode using an ATF system (Mercier et al. 2014; Vellinga et al. 2014), current production of adenoviral vectors (serotype 26 and 35) are carried out only at a PER.C6 cell concentration of about 1.6 × 10^7^ cells/mL (Van and Luitjens 2011).

The theoretical maximum cell concentration, which can be obtained for animal cells, is considered to be about 10^9^ cells/mL (Ozturk 1996). Given that the supply of cells with critical substrates and the removal of growth-inhibiting compounds can always be guaranteed by appropriate feeding and perfusion strategies, the maximum cell concentration largely depends on the volumetric oxygen transfer coefficient (k_L_a) that the cultivation system supports. Accordingly, depending on the cell line, the use of conventional stirred tank or wave bioreactors with k_L_a values up to 55 1/h should allow achieving cell densities in the order of 1 × 10^8^ cells/mL. As expected, experiments show that it is challenging to obtain such high concentration in these cultivation systems and that additional issues, such as accumulation of CO_2_ to toxic concentrations, have to be taken into account. For example, Clincke et al. (2013) have reported previously on CHO cell cultivations exceeding 2 × 10^8^ cells/mL, where a suitable aeration/agitation strategy combined with CO_2_-stripping needed to be implemented.

### Process options to maintain high cell-specific virus yields

To achieve high virus titers, cells should typically be infected during the late exponential growth phase. In addition, an optimal supply of nutrients at toi is required (Aunins 2003). The latter can be achieved by a complete medium exchange prior to addition of the virus seed (Bock et al. 2011; Pohlscheidt et al. 2008) or by an intensive medium renewal during the cell proliferation phase (Thomassen et al. 2014). The use of a perfusion rate of two reactor volumes per day starting immediately after virus infection also helped to improve adenovirus yields in HEK293 cells (Henry et al. 2004). Here, losses of infectious virus particles in the clarified fraction at the early phase of infection were compensated by infecting with an moi two times higher (moi = 20) than in the reference process in batch (moi = 10).

In order to support virus propagation after virus addition, fed-batch mode and/or discontinuous medium exchange have been carried out, especially in processes based on immobilized cells. For example, infecting MCDK cells grown on Cytodex 1, Bock et al. (2011) demonstrated that performing a repeated fed-batch process during the first 2 to 10 h post infection allowed to obtain cell-specific influenza A virus yields threefolds higher compared to a conventional batch process. Similarly, Pohlscheidt et al. (2008) applied a so-called volume-expanded fed-batch strategy during propagation of a *P. ovis* strain in BK cells grown on Cytodex 3. This cultivation strategy consisted in the discontinuous addition of medium to a final volume four times larger than the initial operation volume. Here, total virus yield was increased 40-fold, while virus titers and volumetric productivity were increased in one and two orders of magnitude, respectively, in comparison to a batch process (Pohlscheidt et al. 2008). Compared to a typical fed-batch process (with shorter volume additions), the volume-expanded fed-batch strategy resulted in an almost sixfold increase in total virus yield. Finally, daily harvesting of virus-containing supernatants in a hollow fiber system was reported to produce high titers of influenza A virus and cell-specific virus yields comparable to those obtained in the typical batch production mode in STR (Tapia et al. 2014). This shows that this strategy can be suitable not only for the production of viruses that propagate exclusively in mitotic cells and have a long replication cycle, such as the mink enteritis virus (MEV) (Roya and Mehrad 2008), but also for fast-propagating strains, e.g., influenza A virus.

When operating processes with suspension cell lines, continuous virus harvest/medium exchange is more feasible. In this regard, acoustic filters have been used for cell retention and harvesting of a cell-free virus broth. For example, influenza A virus produced in HEK293 cells was continuously harvested in the clarified supernatant with cell-specific yields of about 4000 virions per cell (Genzel et al. 2014; Petiot et al. 2011). Here, to avoid virus loss in the clarified fraction, medium exchange was not carried out for some hours after infection to allow for an efficient uptake of virions into cells. As addressed before, other commercially available separation systems used at industrial scale, e.g., gravity settlers or spin filters (Pollock et al. 2013), could also allow for continuous virus harvests when infecting suspension cells at concentrations about 2 × 10^7^ cells/mL (Kompala and Ozturk 2006). Furthermore, continuous virus harvests at cell concentrations in the order of 10^8^ could be also possible using new types of bioreactors such as the perfusion bioreactor CellTank®.

An alternative approach to perform virus propagation using perfusion systems is the retention of both cells and virus particles within the bioreactor. For the case of the ATF system mentioned before, it was shown that continuous medium exchange resulted in high cell-specific yields of influenza A virus at laboratory scale. However, the choice of a suitable hollow fiber membrane seems to be a crucial factor since the pore size of membranes seems to have an influence on productivity (Genzel et al. 2014). It is evident that when using membrane-based separation systems, a sound characterization of cell retention during the growth phase must be carried out since any change in porosity and average pore size will have a negative impact on virus retention or harvest titers. Whether it is beneficial to continuously harvest virus particles or to retain them within the bioreactor during the whole virus production phase has to be determined in advance and characteristics of filtration modules have to be chosen accordingly. It might be even beneficial to consider the use of different pore sizes for both cell growth and virus production phases. Similar to acoustic filter-based processes (Genzel et al. 2014; Petiot et al. 2011), for ATF-based processes, a medium exchange should be avoided for few hours after addition of virus seeds to allow for an efficient uptake of virions into cells.

Although ATF and TFF systems share a similar separation principle, i.e., tangential flow filtration, TFF systems have not been used for virus production, so far. One possible reason might be the fact that cells are being exposed to a larger shear stress as TFF systems involve the use of peristaltic pumps (Nienow et al. 2013). It can be expected, however, that improvements such as the implementation of low-stress pumping systems, e.g., magnetic levitated pumps, will promote the use of TFF systems.

### Advantages and challenges of current HCD production processes

Most of the recent approaches toward HCD cultivations in vaccine production have demonstrated that it is possible to maintain or even increase cell-specific virus yields. Given an accumulation of contaminating DNA and host cell proteins proportional to the concentration of cells, the impact on following downstream operations should not be adversely affected. In contrast, the concentration of clarified harvests that is typically performed prior to subsequent chromatography steps either needs only minor modification or, in the best-case scenario, is not required anymore. Furthermore, the use of perfusion systems and a continuous medium exchange during virus propagation phase can mitigate the accumulation of DNA and host cell proteins in the viral harvests. Accordingly, regarding the establishment of HCD cultivations, a negative impact on final product quality is not anticipated.

Nevertheless, there are still various productivity indicators and economic aspects that must be considered, before establishing industrial-scale applications. For example, based on the volumetric productivity (the total amount of virus particles produced per volume of culture medium consumed, virus particles/L), the so-called space time yield (STY, virus particles/(L h)) can be determined taking into account the complete production time. This performance indicator better reflects overall process costs than mere volumetric (virus particles/L) or cell-specific yields (virus particles/cell). This way, even processes with very high product concentration and high cell-specific yield compared to a batch process could be less productive given the large volumes of media consumed or the extended production time or both (Genzel et al. 2014). Also, production process parameters at HCD could impact intrinsic properties, such as the glycosylation of viral proteins and the ratio of infectious virions to the total number of virions, which are key properties of both viral vaccines and viral vectors. Accordingly, small-scale studies addressing medium optimization for HCD cultivations, the establishment of medium feeding, cell cultivation and virus harvesting strategies, and the analysis of critical quality attributes of products are essential to promote the use of HCD processes in routine viral vaccine production.

## Multi-stage bioreactors for continuous virus production

**Fig. 2:**
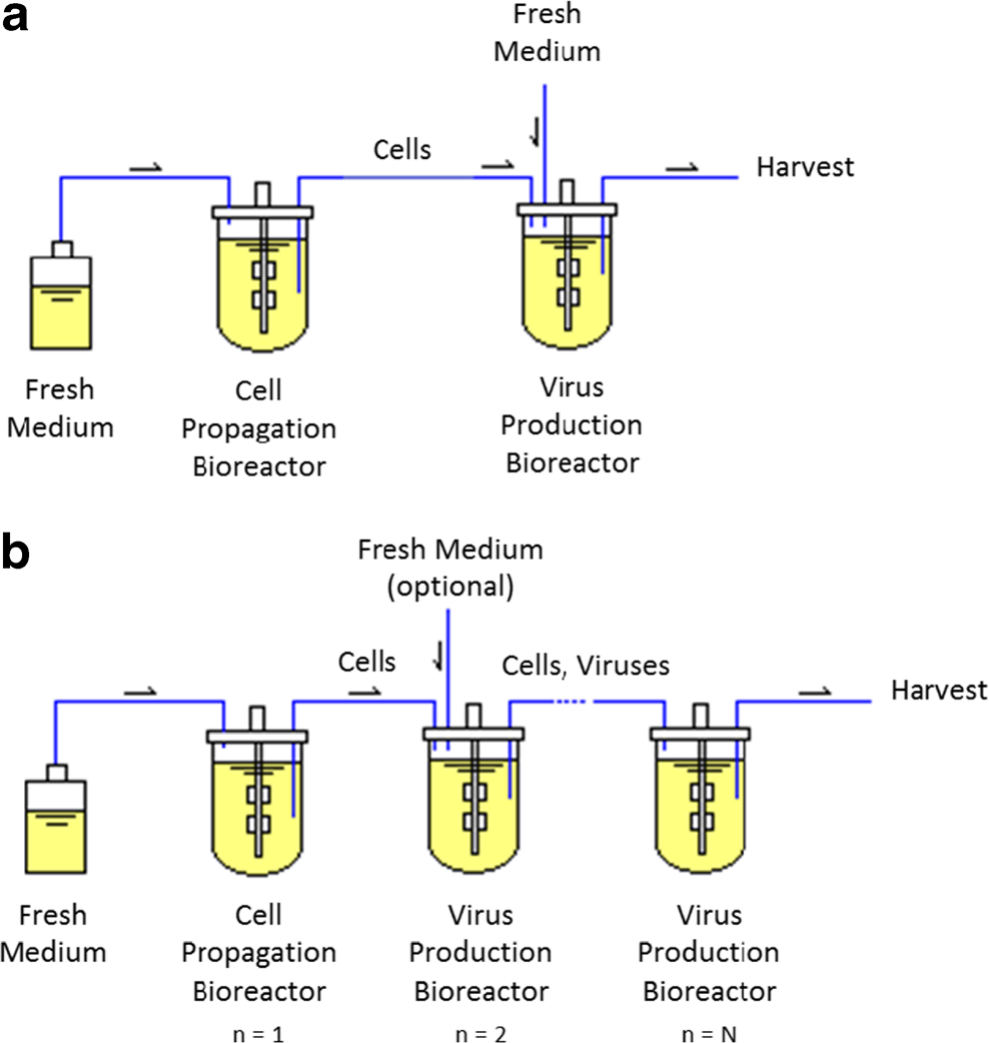
Schematic representations of continuous multi-stage stirred tank reactor (STR) setups. **a** Scheme of a continuous two-stage STR system used for continuous influenza A virus production described by Frensing et al. (2013); **b** a multi-stage STR setup following a plug-flow-like configuration (Hu et al. 1997; Málek and Fencl 1966; van Lier et al. 1990), in which a cascade of STR was used. As before, the first bioreactor (*n* = 1) is exclusively for cell propagation, while virus is produced in the subsequent vessels

The establishment of continuous cultures can be tracked back to the 1950s (Novick and Szilard 1950), where it raised many questions and challenges in the production of biologicals. Finally, however, the focus was on the establishment of batch cultivations due to their advantages regarding ease of operation and process robustness. In addition, productivity increased significantly, as fast advances in genetic engineering were made (Hoskisson and Hobbs 2005). With an ever increasing number of biologicals introduced into the market, however, the interest in more efficient manufacturing platforms is back and tackling several challenges for the next decades, such as the integration of upstream and downstream in fully continuous operated processes, will take a greater role (Warikoo et al. 2012). Continuous production has several well-known advantages compared to the batch cultures, such as steady-state operation, high volumetric efficiency, and lower plant turndown that enhance process yield. Continuous production of many biologicals has been achieved with the use of STR operated in single (chemostat) or multi-stage STR configurations. The use of chemostats (Novick and Szilard 1950) is suitable for molecular biology research or for simple cases, where cells are cultivated on a defined substrate, to obtain maximum biomass and/or high product yields (Málek and Fenel 1966). Nevertheless, stable operation with chemostats can fail, when the product is produced in small amounts (Fencl et al. 1972), when cell growth is inhibited by the product, or in case cell deteriorates as occuring, e.g., with lytic viruses. An alternative to chemostats is multi-stage systems, such as several STRs in series, or STRs in series with tubular bioreactors (Hu and Bentley 2007; Málek and Fencl 1966). One interesting approach for virus production is the use of two-stage STR bioreactors, as depicted in Fig. 2a. Here, the first reactor serves only for cell propagation and the subsequent bioreactor for virus infection and continuous virus replication (Frensing et al. 2013). The addition of more STR in series (Fig. 2b) could potentially increase virus yields by approaching the residence time distribution of a plug-flow reactor or by increasing the RT of cells in the system, with virus release in the subsequent vessels (Gori 1965; van Lier et al. 1990). Therefore, in the following section, the use of multi-stage STR systems using cascades of two and three STR is addressed as an option for process intensification in viral vaccine production.

### Overview of two- and three-stage bioreactors used for continuous production of viruses

Multi-stage systems have been used for propagation of bacteriophages in bacteria (Jacobson and Jacobson 1966) as well as for replication of viruses in human cells (Gori 1965), insect cells (Kompier et al. 1988), and avian cells (Frensing et al. 2013) (see Table 2). Additional options for continuous viral vaccine production exist for processes using persistently infected cells, as reported for instance (Roumillat et al. 1980; Roumillat et al. 1979) for herpes simplex virus growth in lymphoblastoids. Eventually, semi-continuous virus production is also possible in cultures using adherent cells or in cultivation systems involving hollow fiber units. Shen et al. (1996), for instance, used multiple harvest strategies for retroviral production in a NIH 3T3 fibroblast-derived adherent amphotropic murine cell line (pMFG/ΨCRIP) for efficient retroviral production. However, as these options involve only one single stage, where the cell growth phase and the virus replication phase are taking place in the same compartment, they are not considered in this section. Accordingly, in Table 2, only examples for continuous multi-stage production systems established at laboratory scale are summarized.

**Table 2:** List of viruses cultivated in continuous multi-stage bioreactors

Virus	Multi-stage configuration^a^	Cell line	Cell origin	Duration (d p.i)^b^	Max. virus titer (×10^7^ TCID50/mL)	Mathematical model	Passage effect observed?	Comments	Reference
Poliovirus 1	Two STR stages	Hela S-3-1	Human	11	8.3	Yes	No	First concepts and term “lysostat” introduced	Gori 1965
Adenovirus	Three STR stages	Hela-derived KB cell line	Human	6.5	1.0	Yes	No		Gori 1965
Baculovirus E2-strain	Two and three STR stages	Sf-AE-21	Insect	30	1.0^d^	Yes	Yes	Earlier passage effect in three-stage respect to two-stage	van Lier et al. 1990
Recombinant baculovirus—AcMNPV	Semi-continuous repeated fed-batch (two STR stages)	Sf-9	Insect	80	10^c^	Yes	Yes	Passage effect was delayed respect to continuous	van Lier et al. 1996; De Gooijer et al. 1992
Foot-and-mouth disease virus	Semi-continuous two STR stages	BHK21 C13	Mammal	18	1.6	No	No	Bioreactor sizes of 10 and 3 L for cell and virus production, respectively	Roth et al. 1994
Recombinant baculovirus—vIBD-7	STR followed by a tubular reactor (two-stage system)	Sf-9	Insect	8	Not reported	No	Yes	Baculovirus expressing β-galactosidase	Hu et al. 1997
Recombinant baculovirus with extra homologous regions (hrs)	Two STR stages	Se301	Insect	27	100 c	No	Yes	Insertion of an extra homologous region in the BAC vector led to prolonged protein expression	Pijlman et al. 2004
Influenza A/PR/8/34 (RKI)	Two STR stages	AGE1.CR.pIX	Avian	18	700	Yes	Yes	Passage effect led to low yields	Frensing et al. 2013

^a^STR: stirred tank reactor

^b^d p.i: days post infection

^c^Titer of non-occluded viruses (NOVs) in TCID50 per milliliter

^d^Units of polyhedra per cubic centimeter of reactor

### Production of poliovirus and adenovirus

The term “lysostat” was first used to describe a two-stage and three-stage bioreactor for cultivation of poliovirus 1 and adenovirus replicated in a Hela S-3-1 and a Hela-derived KB cell line, respectively (Gori 1965). Poliovirus type 1 was grown with a yield of 421 TCID_50_ per cell and adenovirus type 14 with a yield of 116 TCID_50_ per cell. This pioneer work demonstrated clearly that a continuous production of viruses is possible and introduced a basic mathematical description of two and three continuous STR bioreactors for virus production. It also pointed out that special considerations have to be taken into account for thermolabile viruses. In particular, virus particles have to be removed from the STR with a dilution rate exceeding the specific virus inactivation rate. Furthermore, it is important to keep infected cells in the bioreactor until lysed (or until virus release ceases in case of non-lytic viruses). However, with a life cycle of 5 to 24 h as found for many viruses relevant in vaccine production, this might result in non-optimal steady state conditions and require specific measures to keep virus yields at a high level. Finally, cell concentrations at steady state have to be selected carefully to avoid substrate limitations or the accumulation of inhibiting by-products of metabolism or viral compounds.

### Production of baculovirus

A significant contribution using multi-stage bioreactors and the baculovirus-insect cell expression system was done by the group of Tramper and Vlak (Kompier et al. 1988). In a first publication, two experiments using two-stage cultivation systems operated for 25 and 60 days were described that achieved steady-state production levels of polyhedra and non-occluded virus (NOV) particles for up to 25 days. In this work, for the first time, it was observed that a drop in virus titers is possible at advanced production times (35 days) in continuous mode. And it was suggested that this was due to a “passage effect” induced by DIPs (Krell 1996). A first mathematical model that used a first-order reaction mechanism was introduced later (De Gooijer et al. 1989) to describe baculovirus production in two- and three-stage cultivation systems. The model predicted well the time courses of the viable cell and the non-infected cell concentrations in the virus production bioreactor, but did not describe the passage effect. This last aspect was later covered with a structured model, where the effect of DIPs on virus titers of two- and three-stage STR bioreactors was considered explicitly (De Gooijer et al. 1992). In another publication, a three-stage bioreactor setup (using two vessels for infection) was compared against a two-stage bioreactor system for baculovirus production (van Lier et al. 1990). It was shown that the use of a three-stage bioreactor accelerated the occurrence of viruses with a higher virus passage number, which in turn resulted in an earlier drop in virus yield (passage effect) compared to two-stage cultivations. Thus, three-stage bioreactor setups seem to be disadvantageous for baculovirus production compared to two-stage bioreactor systems as a more plug-flow-like configuration seems to result in viruses with high passage number. In the following, more studies (Lier et al. 1990; van Lier et al. 1992) were carried out using a two-stage bioreactor system to produce a recombinant baculovirus containing the LacZ gene expressing β-galactosidase. For the first time, a DNA analysis showed the existence of a predominant mutant baculovirus that lacked about 40 % of the DNA genome, including the LacZ gene. This confirmed the presence of DIPs in continuous multi-stage baculovirus cultivations and their impact in process productivity. In addition, it allowed to develop hypotheses regarding possible mechanisms of DIP formation (Kool et al. 1991). In another study, baculoviruses were genetically engineered to maintain expression levels (van Lier et al. 1994). However, virus production still decreased after about 30 days of continuous operation. Finally, production in the two-stage reactor system was optimized by performing repeated semi-continuous infections, in which an inoculum of the previous infection was used as seed virus (van Lier et al. 1996). This mode of operation led to an enhancement in performance, compared to continuously operated two-stage systems with regard to longer-term operation. In a more recent study (Pijlman et al. 2004), stability of the virus was increased by the utilization of extra homologous repeat regions, which are located throughout the baculovirus genome and are believed to act as origins of viral DNA replication. This resulted in prolonged protein expression and improved the stability of baculovirus expression vectors for the large-scale protein production in insect-cell bioreactors.

### Production of influenza virus

In a recent study carried out in our laboratory (Frensing et al. 2013), influenza virus A/PR/8/34 (RKI) was continuously produced with the avian cell line AGE1.CR.pIX in a two-stage bioreactor setup operated continuously for 18 days. Virus titers similar to those of batch cultivations published by Lohr et al. (2009) were observed. Unfortunately, virus titers fluctuated over several orders of magnitude due to the presence of defective interfering particles (DIPs), which were confirmed by a PCR assay. A segregated mathematical model of the two-stage system suggested that constant virus titers can only be obtained in the absence of DIPs. Currently, different approaches using the avian suspension cell line AGE1.CR.pIX (Lohr et al. 2014) as a substrate for virus replication are being evaluated in our laboratory to overcome this hurdle for continuous production of influenza virus.

## Outlook on high cell density cultivations and continuous multi-stage bioreactors

It is clear that a long way is still to be passed in vaccine manufacturing to reach the level of process intensification established for other biologicals, i.e., the production of recombinant proteins in CHO cells. Nevertheless, results achieved at the laboratory scale shed a very positive light regarding options for further optimization of large-scale viral vaccine production. Combining knowledge obtained for process intensification in the production of other cell culture-derived biologicals (i.e., the optimization of media, development of designer cell lines, introduction of fed-batch/continuous perfusion systems), advances in automation technologies (on-line monitoring/control of metabolites and cell concentration), and availability of technologies for establishing HCD under cGMP conditions (i.e., membrane-based perfusion systems and hollow fiber units) should allow to catch up fast with the increasing demands for potent and save vaccines at low costs. Continuous processes using multi-stage stirred tank bioreactor systems are also an interesting option to batch production of viruses. In particular, at laboratory scale, experiments have demonstrated that harvests with similar virus titers can be obtained, e.g., for influenza virus production. Duration of continuous processes, however, is clearly limited by the accumulation of DIPs in the population of many (if not most) DNA and RNA viruses. Unfortunately, the presence of DIPs can significantly reduce product titers, and this effect seems to stronger if the number of vessels in a cascade is increased. Thus, the use of two-stage stirred tank bioreactor systems seems to be the best option for intensification of viral vaccine production processes. Furthermore, multi-stage STR systems involving the use of three or more STRs in series would probably not be accepted in large-scale vaccine manufacturing due to the complexity of operation and the increasing risk of process failure. Furthermore, it has to be considered for both two-stage and multi-stage STRs that there is a higher risk of accumulating unwanted antigenic variations of virus strains due to extended process times. Accordingly, all steps towards process intensification have to be carefully evaluated with respect to their potential impact on the quality of the final product, i.e., safety and immunogenicity. Nevertheless, the implementation of HCD cultivations using fed-batch or perfusion strategies seems currently a very attractive option for process intensification. HCD cultivations have the potential to achieve cell concentrations exceeding 10^8^ cells/mL, and it has been shown by several research groups that the so-called cell density effect can be overcome for many viruses. Accordingly, it should be possible to improve productivity in vaccine manufacturing 10- to 100-fold compared to conventional batch cultivations. While it may take several years to translate these ideas into large-scale vaccine manufacturing, HCD cultivations, and for some viruses, cascades of continuous stirred tank bioreactors, are the most promising steps toward manufacturing of more efficacious, safe, and cost-effective viral vaccines.
